# Platelet Extracellular Vesicles Are Taken up by Canine T Lymphocytes but Do Not Play a Role in Their Proliferation, Differentiation and Cytokine Production In Vitro

**DOI:** 10.3390/ijms23105504

**Published:** 2022-05-14

**Authors:** Magdalena Żmigrodzka, Olga Witkowska-Piłaszewicz, Rafał Pingwara, Anna Winnicka

**Affiliations:** 1 Department of Pathology and Veterinary Diagnostics, Institute of Veterinary Medicine, Warsaw University of Life Sciences (WULS-SGGW), 02-787 Warsaw, Poland; anna_winnicka@sggw.edu.pl; 2 Department of Large Animal Diseases and Clinic, Institute of Veterinary Medicine, Warsaw University of Life Sciences (WULS-SGGW), 02-787 Warsaw, Poland; olga_witkowska_pilaszewicz@sggw.edu.pl; 3 Department of Physiological Sciences, Institute of Veterinary Medicine, Warsaw University of Life Sciences (WULS-SGGW), 02-787 Warsaw, Poland; rafal_pingwara@sggw.edu.pl

**Keywords:** extracellular vesicles, exosomes, platelets, lymphocytes, dog, T-cells, B-cells, proliferation, IFN-gamma, IL-17

## Abstract

Eukaryotic and prokaryotic cells in physiological and pathological conditions form membrane-bound extracellular vesicles, known as EVs. The ability of these submicron structures to transfer their cargoes (miRNA, DNA, protein, cytokines, receptors, etc.) into recipient cells is described. Recent data revealed that platelet-derived extracellular vesicles (PEVs) crosstalk promotes cancer growth and metastasis formation. Moreover, they exert immunosuppressive activities on phagocytes. This EV subpopulation is the most abundant amongst all types in circulation. According to the authors’ best knowledge, there is no information regarding the impact of PEVs on canine lymphocytes. The aim of this study was to evaluate the influence of PEVs on lymphocyte proliferation, phenotype and cytokine production in vitro. In the study, it was demonstrated (i) that PEVs interact differently with lymphocyte subsets and are preferentially associated with T-lymphocytes PBMC, while (ii) they are rarely detected in association with B-lymphocytes, and there is evidence that (iii) PEV uptake is observed after 7 h of co-culturing with lymphocytes. In addition, obtained data support the notion that PEVs do not influence in vitro lymphocyte proliferation, differentiation and cytokine production in a canine model.

## 1. Introduction

The number of scientific publications that analyze the role of cell-derived extracellular vesicles (EVs) in pathological processes has risen rapidly in recent years [1,2]. Cell-derived extracellular vesicles (EVs) are secreted by many cells of eukaryote and prokaryote organisms. EVs are defined as a submicron heterogeneous population of spherical cell-derived membrane-enclosed particles [3,4]. They carry a unique content of cargoes from their donor cells, but with no replication ability [2,3]. EVs have been classified into three main groups based upon their size, as exosomes (EXSMs), ectosomes (ECTSMs) and apoptotic bodies (ApBDs). EXSMs are the smallest particles, with a diameter ranging from 30 to 150 nm, ECTSMs between 150 and 500 nm, while the biggest are ApBDs, with a diameter of 800–5000 nm [5,6]. Since 2018, the International Society of Extracellular Vesicles (ISEV) has recommended the classification of EVs as small, with a diameter below 200 nm, and medium or large with their diameter over 200 nm [3]. In EV classification nomenclature, as important as their size is their cellular origin [7]. EV biogenesis is not accidental and EXSMs are constructively generated from late endosomes, ECSMs are produced from budding cell membranes, and ApBDs are released during apoptosis [5,8]. Cell proliferation, maturation or their aging, as well as the cellular stress response, lead to the shedding of EVs into extracellular space [9].

The cargoes of EVs vary between EXSMs, ECTSMs and ApBDs and reflect the intracellular origin of the cell type from which they are derived. EVs contain about 70% of parental glycoproteins, maternal surface receptors (e.g., CD61- platelets, CD3-lymphocytes T), phosphatidylserine, heat shock proteins (HSPs), tetraspanins, enzymes (aldolase, matrix metalloproteinases), mRNA, miRNA and other biologically active molecules [10]. These functional cargoes determine the impact of EVs on recipient cells. Newly generated EVs are secreted into extracellular space, but they are also present in blood and other body fluids [11].

EVs have been successfully isolated from different body fluids: breast milk, synovial or cerebral fluid, urine and others. Nevertheless, the volume of the specimen and the procedure of EV isolation could provide some methodological difficulties [12]. Peripheral blood samples seem to be the foremost and most-often-collected source of EVs for scientific and clinical research in human medicine. The most abundant population of EVs in the blood of healthy subjects, ranging between 70 and 90%, are platelet-derived EVs (PEVs) [13,14].

PEVs, as subcellular material in plasma and serum, described as “platelet dust”, were reported for the first time in 1967 by Peter Wolf [15,16]. They are secreted throughout platelet (PLT) activation and are known to be a pivotal factor in coagulation and clot formation [13,15,17]. The number of PEVs fluctuates depending on different physiological and pathological conditions [18,19,20]. Their number increases in cancer up to four times compared to healthy individuals [21,22]. Moreover, PEVs’ involvement in cancer, cardiovascular and autoimmune diseases has been reported [4,7,9,18,21]. Every year, an increasing number of papers report the role of PEVs in cancer progression and show their elevated number as a useful prognostic factor in cancer [22,23,24,25,26]. Thus, it is interesting that PEVs may have an influence on peripheral blood lymphocytes as well. In the burgeoning world of microvesicle biology, their role in the cellular material exchange among a variety of cells is currently generating a growing interest in the scientific community. Numerous papers show that tumor-derived EVs (TEVs) are implicated in modulating the tumor microenvironment (TEM), acting as immunomodulators and contributing to the inhibition of anti-tumor activity. They act by horizontal transfer of their cargo into TEM cells, including lymphocytes, NK cells, endothelium cells or monocytes [27,28,29,30]. PEVs, as the most abundant amongst all types of circulating EVs in healthy individuals and during different pathologies, appear to be equally important in cell-to-cell communication. Their role in cancer angiogenesis, progression and metastasis is well documented [31,32,33,34]. Moreover, their significant role in coagulation hemostasis and inflammation has been shown [35,36,37,38]. PEVs increase PGI2 production and expression of surface molecules CD11a, CD14 and MAC-1 on monocytes and enhance their adhesion to endothelial cells [18,35]. The presence of PEVs with P-selectin in the inflammatory milieu is a pivotal element of neutrophil aggregation and accumulation [13,18,35].

In previous work, we showed that PEVs are present in canine plasma and their absolute number significantly increased in animals with neoplasm. Moreover, similarly to humans, PEVs made up the largest population of EVs [39]. As PEVs are important mediators in cell-to-cell communication, we decided to examine their influence on peripheral blood mononuclear cells (PBMCs) in healthy individuals. However, according to the authors’ best knowledge, there is no information regarding PEV impact on dog lymphocytes.

## 2. Results

### 2.1. Platelet-Derived Extracellular Vesicles (PEVs) Are Taken up By Lymphocytes

**Figure 1:**
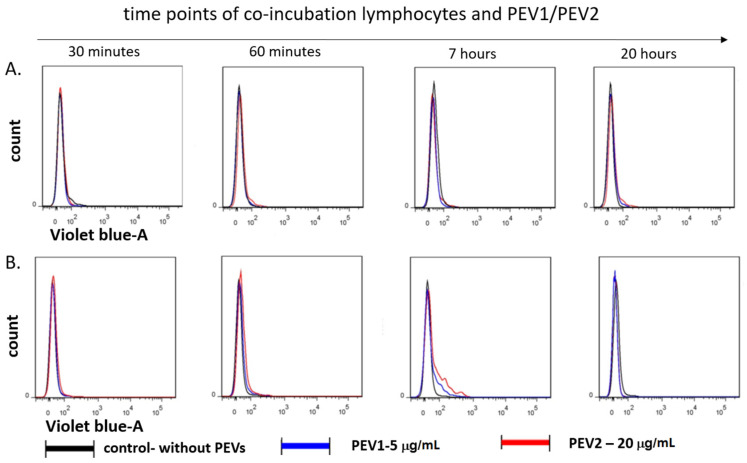
Flow cytometric analysis of the fluorescence transferred by PEVs into lymphocytes. The staining of directly labeled cells and the negative control cells (gray line) after 30 min, 7, and 20 h of incubation are shown. (**A**) The kinetics of PEVs uptaken by B CD21^+^ lymphocytes and (**B**) PEVs uptake by T CD3^+^ Lymphocytes.

To demonstrate the uptake of PEVs by target cells, 5 × 10^5^ PBMCs were exposed for 0.5, 1, 7 and 20 h to CellTrace Violet-labeled PEVs at a concentration of PEV1 (5 μg/mL) or PEV2 (20 μg/mL) in 0.5 mL medium. Cells were incubated at 37 °C with 5% CO_2_ in the presence of ConA (5 μg/mL). After the incubation, the cells were washed in dPBS, stained with mAbs, and immediately analyzed by flow cytometry. The results confirmed that PEVs are taken up by T lymphocytes (Figure 1). The PEV uptake was not observed within 1 h but it was detected after 7 h of co-incubation. After 20 h, PEV fluorescence was not observed on lymphocytes.

### 2.2. PEV Interactions with Lymphocyte Subsets, Their Proliferation and Cytokine Production

To investigate the potential impact of uptaken PEVs on T-cell function, we co-incubated PBMCs activated by ConA and IL-2, with the presence of two PEV concentrations. After 5 days, the intracellular release of specific T-cell cytokines IFN-γ and IL-17 was analyzed.

#### 2.2.1. PEV Does Not Influence Lymphocyte Proliferation

**Figure 2:**
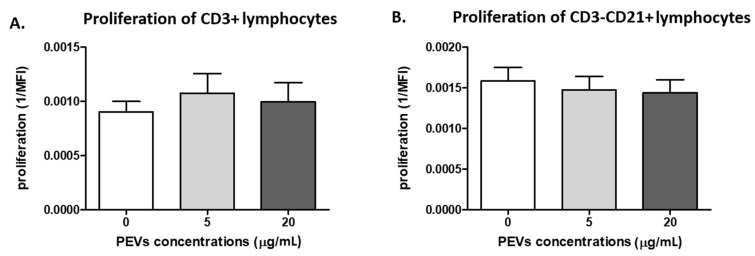
The figures show the proliferation intensity of CD3^+^ (**A**) and CD3^-^CD21^+^ (**B**) lymphocytes after 5 days of culture of PBMC in a 37 °C, 5% CO_2_ environment with ConA and IL-2 presence and PEV1/PEV2 or without PEVs (*n* = 8). The results are presented as the mean ± SEM.

There were no differences in proliferation of PBMCs in the presence of PEVs compared to samples stimulated only by ConA and IL-2 (*p* > 0.05). Proliferation of T-lymphocytes (CD3^+^) was found in the control samples (0.0009), PEV1 (0.0010) and PEV2 (0.0009). For B-cells (CD21^+^), proliferation was 0.0015, 0.0014 and 0.0014 (Figure 2A,B).

#### 2.2.2. PEV Has No Impact on Lymphocyte Immunophenotype

**Figure 3:**
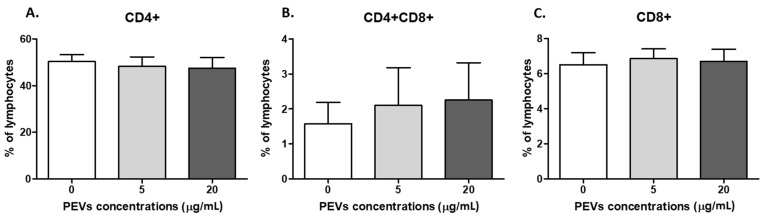
The percentage of CD4^+^ (**A**), CD4^+^CD8^+^ (**B**) and CD8^+^ (**C**) lymphocytes after 5 days of culture of PBMC in a 37 °C, 5% CO_2_ environment with ConA and IL-2 presence and PEV1/ PEV2 or without PEVs (*n* = 8). The results are presented as the mean ± SEM.

There was no PEV influence on T lymphocytes immunophenotype (*p* > 0.05). The CD4^+^cell percentage was 50.3 ± 8.5% in the control, 48.2 ± 11.6% with PEV1 and 47.5 ± 12.7% for PEV2, respectively (Figure 3A). The percentage of double-positive cells (CD4^+^CD8^+^) was 1.5 ± 1.7%, 2.1 ± 3.1% and 2.25 ± 3.0% (Figure 3B) and for CD8^+^ lymphocytes, 6.5 ± 1.9%, 6.8 ± 1.5% and 6.6 ± 2.0%, respectively (Figure 3C).

**Figure 4:**
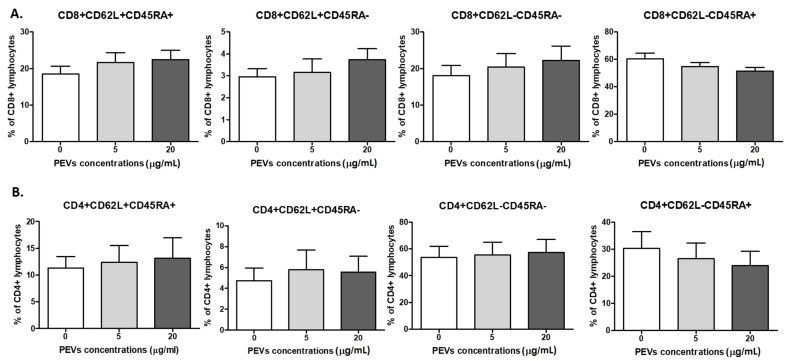
Changes in frequencies of canine CD8^+^ (**A**) and CD4^+^ (**B**) lymphocytes after 5 days culture of PBMCs in a 37°C, 5% CO_2_ environment with ConA and IL-2 presence and PEV1/ PEV2 or without PEVs (*n* = 8). The results are presented as the mean ± SEM.

Further, there was no PEV influence on the percentages of CD4^+^ and CD8^+^ naïve, central memory (CM), effector memory (EM), and terminally differentiated effector memory (TEMRA) cells (*p* > 0.05). Their phenotype was as follows: naïve (CD45RA^+^CD62L^+^), CM (CD45RA^-^CD62L^+^), EM (CD45RA^-^CD62L^-^), TEMRA (CD45RA^+^CD62L^-^). There was no change in the percentage of naïve Th-lymphocytes in the control sample 11.3 ± 2.1%, PEV1 12.3 ± 3.1% and PEV2 13.1 ± 3.8%. The percentages of Th CM cells were 4.7 ± 1.2% in the control sample and 5.7 ± 1.8% and 5.6 ± 1.5% for PEV1 and PEV2 EM cells. The most numerous were EM cells: in the control sample 53.6 ± 8.2%, for PEV1 55.4 ± 9.5% and PEV2 57.4 ± 9.7%; the TEMRA percentages were as follows: 30.3 ± 63%, 26.4 ± 5.8% and 23.9 ± 5.2% (Figure 4A).

The percentages of Tc naïve cells were: 18.5 ± 2.1%, 21.6 ± 2.7% and 22.5 ± 2.5%, for CM 2.9 ± 0.4%, 3.2 ± 0.6% and 3.7 ± 0.5%, respectively. For EM cells, the percentages were: 18 ± 2.8% and 20.4 ± 3.6% and 22.2 ± 3.9%; for TEMRA cells, respectively: 60.6 ± 4%, 54.8 ± 3% and 51.6 ± 2.7% (Figure 4B).

#### 2.2.3. PEV Does Not Influence Lymphocytes T Cytokine Production

**Figure 5:**
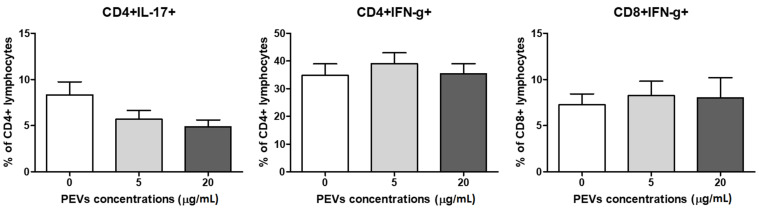
Changes in frequencies of canine CD4^+^IFNγ^+^ and CD8^+^IFNγ^+^ and CD4^+^IL17^+^ lymphocytes after 5 days of culture of PBMCs in a 37 °C, 5% CO_2_ environment with ConA and IL-2 presence and PEV1/ PEV2 or without PEVs (*n* = 8). The results are presented as the mean ± SEM.

The intracellular production of IFNγ by CD4^+^ and CD8^+^ lymphocytes was evaluated (Figure 5). There was no PEVs influence on IFNγ production (*p* > 0.05). In control cells, the percentages of IFNγ^+^ cells were 34.8 ± 4.1% and 7.2 ± 1.2%. After stimulation of PEV1, the percentages were 39.0 ± 3.9% and 8.2 ± 1.6% and in the presence of PEV2, the percentages were 35.4 ± 3.7% and 8.0 ± 2.1%, respectively, for CD4^+^ and CD8^+^ lymphocytes. Further, intracellular expression of IL-17 was estimated for CD4^+^ cells and 8.3 ± 1.4% and 5.7 ± 0.9% and 4.8 ± 0.7%.

## 3. Discussion

First of all, concentration and PEV cargoes can be affected by such factors as: collection method, type of chosen anticoagulant or sample storage. These may lead to the formation of heterogeneous PEV populations with different cargoes (e.g., protein profile), which affect their role in cell-to-cell communication [18,40]. The choice of anticoagulant is crucial to avoid PLT activation during the collection and storage of blood products (whole blood or PRP). Jayachandran et al. examined the influence of selected anticoagulants on the quantity of PEVs and endothelium-derived EVs in healthy individuals. They chose the most common anticoagulants: ethylenediaminetetraacetic acid (EDTA), Na citrate, acid citrate dextrose (ACD solution B) and sodium heparin [39,41]. The number of PEVs was significantly lower in the samples containing calcium-chelating anticoagulants (EDTA, Na citrate, ACD) compared to protease inhibitors (heparin) [41]. A similar observation was made by Weiss et al., in citrated and heparinized blood and platelet-free plasma (PFP) in humans [42]. In the study, the PEVs isolated from blood collected at the EDTA anticoagulant.

Despite a previously documented lower influence of EDTA at lower PEV concentrations, it has the best impact on PEVs stability during storage. It was documented that during the storage of whole blood or platelet concentrates, the number of erythrocyte-derived EVs and PEVs increases [43,44]. In another study, the PEV count in EDTA was stable for 48 h [45]. PLTs have a short life span, approximately 5 to 7 days; moreover, the ex vivo activation of PLTs in the PRP and their storage lesion with apoptotic EV release are time dependent. They could increase PEV number in PRP [46,47]. Vasina et al. showed that PEVs released during storage periods express activated IIb3 integrins and tend to assemble into aggregates [48]. In contrast, PEVs formed after thrombin or Ca2+ ionophore PLTs activation had non-activated αIIbβ3 and increased CD63 surface expression. Regardless of PLT activator type, PEVs express CD62P, which is crucial for their interaction with monocytic cells, but the changes in their functions differ depending on various PLT activators [48]. Acquainted with that, we decided to store PRP only for 24 h preceding PEVs isolation, to avoid the impact of PEVs formed during storage on examined cells.

Moreover, several studies have been performed, comparing the effect of different PLT activators on differences in PEV release [49]. Aatonen and her colleagues showed a list of diminishing PLT activators: Ca++ ionophore > thrombin > CRP-XL > TC co-stimulus > collagen > LPS > TRAP-6 > ADP [50]. These observations were consistent with previous studies [18,51,52]. Agonist stimulation enhanced PEV release and led to lower negatively charged PS externalization in PEVs, compared to those generated without agonist stimulation [53]. Phosphatidylserine (PS) exposure on PEVs during their formation from PLTs is crucial for their physiological procoagulant activity. In EV phenotyping, annexin V (ANX V) and lactadherin (LA) are used as markers of PS [39,42,54]. PS expression on the outer-membrane surface of apoptotic cells is an “eat-me” signal for macrophages and other phagocytic cells. Interestingly, in LA–deficient mice, an increased level of PEVs in the bloodstream is observed, where the level indicates that LA may also play a role in the clearance of PS-expressing PEVs from the circulation, thus, reducing the hypercoagulable state [55,56]. PS, as a key structural element on apoptotic cells, when expressed on PEVs, could be the main signal of preferential uptake of PEVs by monocytes.

Rolling the stored blood before PEVs isolation strongly increases the release of PEVs when a citrate anticoagulant is used, whereas the number of PEVs is stable in EDTA samples [57]. Moreover, the number of PEVs and erythrocyte-derived EVs is persistent at RT for 48 h in EDTA blood samples, but the procoagulant activity of EVs increased after 8 h of storage [44]. The mechanism by which PEV formation is induced is critical for their phenotype and function. It shows that the recommended anticoagulant for PEV isolation should be chosen based on the experimental purpose. In our work, obtained PEVs were obtained from EDTA PRP after 24 h of rotation, due to the fact that PLT activation and then PEVs formation during storage were limited.

In addition, there are numerous studies showing that PEVs incorporation by cells depends on culturing duration or cell type. Data shown by Dinkla et al. and Sadallah et al. demonstrate PEVs corporation after co-culturing them with T cells within 17 to 20 h [58,59]. In mentioned studies, the source and storage time of PEVs were different. In a study by Dinkla et al., PEVs were isolated from ACD platelet-rich plasma (PRP), obtained from healthy donors and stored for 7 days at RT in a lateral motion. To obtain PEVs, Sadallah et al. used platelet concentrate stored at RT, in motion for 5 days with trisodium citrate and an additional T-Sol mixture [58,59]. Then, the interactions between CD4^+^ and CD8^+^ lymphocytes and PEVs were observed for PEVs concentration from 5 to 15 µg/mL or when 1.5 × 10^6^ Tregs were co-cultured with PEVs for 16 h [58,59]. Accordingly, in the current study, it is demonstrated that PEVs are uptaken by lymphocytes within 7 h of co-incubation, but not after 20 h. However, the opposite observation was made by Weiss et al., who showed preferential PEVs internalization by monocytes, while B cells, T cells and NK cells remained free from PEVs [42]. In that work, to characterize the PEVs association with leukocytes, the tests were made in whole blood. In the author’s opinion, during PEVs isolation procedures (e.g., centrifugation), the number of PEVs would be depleted. The scarcity of PEVs fusion with B and T lymphocytes was observed after 3 h of co-incubation, which was in contrast to favored monocyte uptake at the same time point [42]. In our study, the PEVs were incorporated only by T lymphocytes, thus, we evaluated only PEVs influence on the cells. The T cells uptake PEVs within 7 h, but not after 20 h. We consider that PEVs with their expression of phosphatidylserine were effectively taken up an phagocyted by monocytes in cell culture, which was observed by Weiss et al. In contrast, in Sadallah’ work, PEVs were detected on CD4 + T cells after 20 h of incubation only CD4+ and CD8 + T cells subsets [49,52].

The EV phenotype is characterized by the presence on their surface of typical maternal receptors [60]. The most common integrin receptor αIIβ3 present on PLT surfaces was favored as a PEV marker. Increasingly, CD41-labeled EVs are accepted as PEVs, as well as megakaryocyte-derived EVs (MkEVs). New data show that CD41^+^ EVs in blood could be produced by bone marrow megakaryocytes, whereas the PEVs should be described as CD62P^+^ or CD41^+^CD62P^+^ [18,58]. However, the number of CD62P^+^ EVs increases in pathological conditions in blood and during the storage of PRP as well [18,58,61]. In both works, Dinkla et al. and Sadallah and colleagues, the PRP was the PEV source and was stored in RT for 5 to 7 days. In these conditions, the isolated PEVs were mainly from activated and aging PLTs. After co-culturing Treg cells with PEVs, the authors demonstrated that they prevent differentiation of peripheral blood-derived Tregs into IL-17– and IFNγ–producing cells [58]. This work proves that PEV binding changes IL-17’s producing capacity by memory-like Tregs (CD41^+^CD25^high^Foxp3^+^). In our work, there were no differences in IL-17 and IFN-γ production in lymphocytes after PEVs co-culturing. Furthermore, it is interesting that about 8% of Tregs in healthy humans express CD41 and CD62P on their surface. That indicates that CD62P^+^ PEVs could also be present in healthy individuals and it needs further investigation [58]. In another study, it was shown that exposure of activated CD4^+^ T cells to PEVs decreased their release of several cytokines, such as IFNγ, TNFα and IL-6, and increased the production of TGF-β1 and increased frequencies of CD25^high^ Foxp3^+^ cells [59]. Moreover, it shows that PEVs induce differentiation of CD4^+^ T cells towards functional Tregs and this may represent a mechanism by which PEVs enhance peripheral tolerance [59]. Moreover, PEV-treated Foxp3^+^ cells were as effective as peripheral blood Tregs in suppressing CD8^+^ T-cell proliferation. Thus, the potential role of PEVs from blood products as immunosuppressive components was confirmed. In contrast, in the present study, there was no increase in proliferation rate and no changes in phenotype or cytokine production after co-culturing with similar concentrations of PEVs as in Sadallah’s work. The possible reason for our observations being contrary to Dinkla et al. and Sadallah could be connected with several differences in storage. In the two previously mentioned studies, time of storage for PRPs was longer than in our experiment, which could reflect the different PEV cargo and result in a different biological effect.

Study limitations: Based on good ethical practices in executing experiments in animal models, the study was not performed in vivo, which is the main limitation of the study. In addition, in that study, PEVs were formed only during a short storage period of PRP from one patient and were used to perform all in vitro experiments (controls and examined: PEV1 and PEV2), with PBMCs from a limited number of dogs. We decided to use young adult dogs, not older patients, to avoid the possibility of systemic diseases. In addition, actions had to be taken to obtain as accurate results as possible during the experiments.

## 4. Materials and Methods

### 4.1. Animals and Blood Samples

Ten adult dogs, presented for periodic health examination to a veterinary clinic in Warsaw, were included in the study. The qualifying criteria were no clinical signs of disease during anamnesis, and clinical examination. The inclusion criteria were no vaccination or treatment two weeks before blood sampling. The median age of the dogs was 3.5 years (range 1–6). There were six females (two neutered) and four males. Two mixed breeds and Greyhounds, and one Border Terrier, Whippet, Beagle, Great Dane, Standard Schnauzer and Swiss Shepherd.

Only excess peripheral blood (less than 3 mL) collected for routine diagnostic tests was used for this study. As blood collection was a part of a non-experimental clinical veterinary examination consented to by the owners of dogs, according to the European directive EU/2010/63 and local regulations regarding animal experiments, there was no need for the approval of the Ethical Committee.

The dogs were required to fast for routine blood sampling although fasting was not required specifically for the purpose of this study. Peripheral blood samples were taken by cephalic or saphenous venipuncture after 12 hours of fasting. Blood was anticoagulated with dipotassium ethylenediaminetetraacetic acid (K2-EDTA). Hematological analysis was performed on all dogs as a part of their initial evaluation. A complete blood count was done (ProCyte DxHaematology Analyser, IDEXX, Westbrook, ME, USA), and blood smears were examined with a CX21 light microscope (Olympus, Tokyo, Japan) after May-Grünwald Giemsa staining. The excess amount of blood samples used for hematology testing was utilized to determine the influence of PEVs on peripheral blood mononuclear cells (PBMC) in culture. Blood smear examination and cell culture were performed at the Department of Pathology and Veterinary Diagnostics at the Institute of Veterinary Medicine, Warsaw University of Life Sciences (WULS-SGGW), Warsaw, Poland.

### 4.2. PEV Isolation and Staining

The platelet-rich plasma (PRP) was estimated from fresh K2-EDTA whole blood by centrifugation 300× *g* 10 min at RT (MPV-260R; MPW med. instruments, Warsaw, Poland) [62,63], maintaining sterile conditions. Next, the upper half (an aliquot of 2 mL) of the supernatant was carefully pipetted and stored in motion for 24 h in a Multi-Rotator at RT (Mukti Bio RS-24bioSan, Riga, Latvia). Then, the modified centrifugation protocol for PEV isolation was done [59]. To remove any residual leukocytes or erythrocytes the samples were centrifuged for 15 min at 500× *g*. Following that, the supernatant was centrifuged 800× *g* for 20 min to pellet PLTs. To eliminate any residual PLTs and low-density debris an additional centrifugation step-3000× *g* 20 was performed. To pellet PEVs, the supernatant was carefully transferred to another tube and centrifuged 22,000× *g* for 45 min [58]. Subsequently, the pellet was aliquoted in dPBS and frozen at −80 °C until use. All centrifuge steps were made at 4 °C. Extracellular vesicle protein concentration was determined by a BCA Pierce™ BCA Protein Assay Kit (Thermo Fisher Scientific, Waltham, MA, USA; 23225) according to the manufacturer’s protocol. Absorbance at 562 nm was measured by BioTek Synergy H1 multiplate reader (BioTek Instruments GmbH, Bad Friedrichshall, Germany).

To evaluate PEV uptake by PBMCs, PEVs were labeled with a 5 μM CellTrace Violet cell proliferation kit (Life Technologies, Bleiswijk, The Netherlands) for 30 min at RT, away from the light. To eliminate unincorporated dye, one centrifugation step (22,000× *g* 45 min at 4 °C) was completed [64,65]. Next, PEV pellets were resuspended in the completed cell medium and were added to PBMCs. The cells were incubated at 37 °C with 5% CO_2_ and harvested after 30 min, 1, 7 and 20 h. Finally, the cells were stained with mAbs as described above.

### 4.3. Cells Isolation and Culture

The cells were isolated from fresh K2-EDTA whole blood by density gradient centrifugation, maintaining sterile conditions. Histopaque 1077 (Sigma-Aldrich, Germany) was used for the separation of peripheral blood mononuclear cells (PBMCs), according to the manufacturer’s recommendations: about 3 mL of blood, gently mixed with 3 mL of buffer (dPBS) (Gibco, Life Technologies, Bleiswijk, the Netherlands) at RT, was layered on 3 mL of Histopaque 1077 in a sterile, V-bottom tube and then centrifuged (400× *g*) for 25 min at RT, without a brake (MPV-260R; MPW med. instruments, Warsaw, Poland). The collected fraction of PBMC was then washed with RPMI 1640 with GlutaMAX TM (Gibco, Life Technologies, Bleiswijk, the Netherlands), followed by centrifugation for 5 min at RT (400× *g*) and resuspended in 2 mL of completed medium: RPMI 1640 with GlutaMAX™ (Gibco, Life Technologies, Bleiswijk, the Netherlands) containing 10% heat-inactivated fetal bovine serum (FBS), penicillin (100 IU/mL), streptomycin (100 μg/mL), nonessential amino acids (1%), MEM vitamins (100 μM), sodium pyruvate (1 mM) and amphotericin B (1 μg/mL) (Gibco™, Life Technologies, Bleiswijk, the Netherlands). The following day, sample cellularity and cell viability were assessed in an EVETM cell counter (NanoEntek, Seoul, Korea). Next, 2 × 10^6^ freshly isolated PBMC was cultured in the absence or presence of PEV1 (5 μg/mL) or PEV2 (20 μg/mL) with concanavalin A (ConA) (Sigma-Aldrich, St. Louis, MO, USA; 5 μg/mL). After 24 h the cells were washed and recombinant canine IL-2 (R and D Systems, Abingdon, UK; 1 ng/mL) was added, and then the cells were incubated for another 4 days. All the cells were incubated at 37 °C with 5% CO_2_. The cultured cells were then used to evaluate the influence of PEV1 and PEV2 on the proliferation of lymphocytes, as well as antigen expression and intracellular cytokine production. On the fifth day the cells were restimulated with phorbol 12-myristate 13-acetate (PMA) and ionomycin (eBioscienceTM Cell Stimulation Cocktail, InvitrogenTM, Waltham, MA, USA; 5µg/mL). In addition, samples for cytokine production were incubated with BD GolgiStop Protein Transport Inhibitor with Monensin (BD, Franklin Lakes, NJ, USA) in sterile conditions at 37 °C with 5% CO_2_.

### 4.4. Cell Staining

Samples with suspended cells intended for the determination of cell proliferation were supravitally stained with CellTrace™ Violet Cell Proliferation Kit (Life Technologies, Bleiswijk, the Netherlands) before ConA stimulation and PEV1 or PEV2 supplementation culturing according to the manufacturer’s instructions.

Lymphocytes were characterized by examination of the expression of the surface markers using canine-specific monoclonal antibodies (mAbs) or with documented cross-reactivity (included in Table 1).

The appropriate amount of each mAb was determined experimentally to obtain optimal labeling results. The controls included unlabeled cells, and when necessary, FMO (fluorescence minus one) controls were used. For blocking of nonspecific mAbs binding, 10% BSA (15 min at 4 °C) before staining with antibodies was used. The cells were incubated with antibodies for 20 min at RT in eBioscience™ Flow Cytometry Staining Buffer (Life Technologies, Bleiswijk, the Netherlands) and protected from light. The cells were then washed twice with 2% BSA and resuspended in a 200 μL flow cytometry staining buffer, and immediately introduced into the cytometer.

For intracellular staining of IL-17 and IFNγ, after surface mABs staining, cells were incubated with the permeabilization solution (20 min at RT in dark) (BD Cytofix/Cytoperm™, BD, USA). Next, after the washing step, the cells were incubated with IL-17 and IFNγ mAb (30 min at 4 °C), washed, and then resuspended with a 200 μL flow cytometry staining buffer. For tubes with mAbs -CD45RA, a two-step staining procedure was used. After the washing step with 2% BSA, the cells were incubated with IgG1 conjugated with PE (20 min at 4 °C). Lastly, the cells were washed with 2% BSA and resuspended with a 200 μL flow cytometry staining buffer, and immediately introduced into the cytometer.

### 4.5. Flow Cytometry Analysis

**Figure 6:**
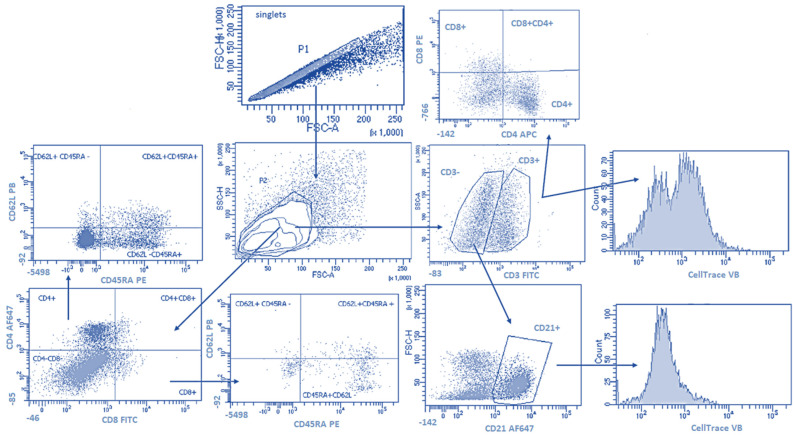
The gating strategy. Doublets were removed from the analysis by setting the gate on single cells on the FSC-area (FSC-A) vs. FSC-high (FSC-H) dot plot (P1). Next, the lymphocytes were gated based on FSC and SSC dot plots (P2). Then cell proliferation calculated from the gate included lymphocytes as CD3^+^ (lymphocytes T) and cells CD3^-^ and CD21^+^ (lymphocytes B). Next, the sample included CD4^+^ and CD8^+^ cells with co-expression of CD62L and CD45RA was analyzed.

The gating strategy is shown in Figure 6. Doublets were removed from the analysis by setting the gate on single cells on the FSC-area (FSC-A) vs. FSC-high (FSC-H) dot plot. Cell proliferation was calculated from singlets for lymphocytes T (CD3^+^) and B (CD3^-^ CD21^+^). Next, the lymphocytes were gated based on FSC and SSC dot plots. In the T-lymphocytes gate analyses of CD4^+^ and CD8^+^ cells with co-expression of CD45RA and CD62L were performed. The third and fourth sample included CD4^+^ and CD8^+^ cells with co-expression of intracellular IFNγ and IL-17. A flow cytometric analysis was performed using a FACSCanto II flow cytometer and Diva software (Becton Dickinson, Franklin Lakes, NJ, USA); 20,000 cells of each sample were acquired. Prior to multicolor staining, the compensation was set using single-positive cells for each color.

### 4.6. Statistical Analysis

Statistical analysis was performed in Prism software, version 5.0 (GraphPad Software, San Diego, CA, USA). One-way ANOVA and Tukey’s HSD post hoc test were applied to determine the statistical significance of control cells (not PEV treated) and PEV-treated cells between different concentrations of the PEVs. On the assumption that *p*-value < 0.05 was regarded as significant, *p*-value < 0.01 and p-value < 0.001 were highly significant. In our study, we did not observe significant changes between non-treated and PEV-treated samples.

## 5. Conclusions

PEVs are the most abundant amongst all types of EVs in circulation. However, the mechanisms leading to PEV release and phenotypic composition are insufficiently understood. According to the authors’ best knowledge, there is no information regarding the impact of PEVs on canine lymphocytes. In conclusion, we demonstrate (i) that PEVs interact differently with lymphocytes subsets and are preferentially associated with T-lymphocytes in canine PBMCs, while (ii) they are rarely detected in association with B-lymphocytes, and we provide evidence that (iii) PEV uptake is observed after 7 h of co-culturing with lymphocytes. Moreover, our data support the notion that PEVs do not influence lymphocyte proliferation, differentiation and cytokine production in vitro.

## Figures and Tables

**Table 1:** List of monoclonal antibodies used for labeling peripheral blood mononuclear cells (PBMCs) for flow cytometry. Abbreviations: FITC—Fluorescein isothiocyanate, PE—Phycoerythrin, AF647—Alexa Fluor 647, PB—Pacific Blue.

Antibody	Clone	Isotype	Host Species	Fluorochrome	Catalog Number	Supplier	Dilution
CD3: CD8	CA17.2A12/ YCATE55.9	IgG1	mouse	FITC:PE	DC047	BioRad	1:5
CD4: CD8	YKIX302.9/ YCATE55.9	IgG2a	rat	FITC:PE	DC048	BioRad	1:5
CD4	YKIX302.9	IgG2a	rat	AF647	MCA1038A647	BioRad	1:5
CD8	YCATE55.9	IgG1	rat	FITC	MCA1039F	BioRad	1:5
CD21	CA2.1D6	IgG1	mouse	AF647	MCA1781A647	BioRad	1:5
CD62L	FMC46	IgG2b	mouse	PB	MCA1076PB	BioRad	1:5
CD45RA	CA4.1D3	IgG1	mouse	-	MA5-16612	Invitrogen	1:5
IgG1	M1-14D12	IgG1	rat	PE	12-4015-82	Invitrogen	1:5
IFNγ	CC302	IgG1	mouse	AF647	MCA1783A647	BioRad	1:5
IL-17A	eBio17B7	IgG2a,κ	rat	PE	12-7177-81	Invitrogen	1:20

## Data Availability

The data presented in this study are available on request from the corresponding author.
